# Microevolution of Anthrax from a Young Ancestor (M.A.Y.A.) Suggests a Soil-Borne Life Cycle of *Bacillus anthracis*

**DOI:** 10.1371/journal.pone.0135346

**Published:** 2015-08-12

**Authors:** Peter Braun, Gregor Grass, Angela Aceti, Luigina Serrecchia, Alessia Affuso, Leonardo Marino, Stefania Grimaldi, Stefania Pagano, Matthias Hanczaruk, Enrico Georgi, Bernd Northoff, Anne Schöler, Michael Schloter, Markus Antwerpen, Antonio Fasanella

**Affiliations:** 1 Bundeswehr Institute of Microbiology, Munich, Germany; 2 Technische Universität München, Wissenschaftszentrum Weihenstephan, Chair for Soil Ecology, Freising, Germany; 3 Istituto Zooprofilattico Sperimentale of Puglia and Basilicata, Anthrax Reference Institute of Italy, Foggia, Italy; 4 German Research Center for Environmental Health, Research Unit for Environmental Genomics, Neuherberg, Germany; 5 Ludwig Maximilians Universität München, Institute for Laboratory Medicine, Munich, Germany; ContraFect Corporation, UNITED STATES

## Abstract

During an anthrax outbreak at the Pollino National Park (Basilicata, Italy) in 2004, diseased cattle were buried and from these anthrax-foci *Bacillus anthracis* endospores still diffuse to the surface resulting in local accumulations. Recent data suggest that *B*. *anthracis* multiplies in soil outside the animal-host body. This notion is supported by the frequent isolation of *B*. *anthracis* from soil lacking one or both virulence plasmids. Such strains represent an evolutionary dead end, as they are likely no longer able to successfully infect new hosts. This loss of virulence plasmids is explained most simply by postulating a soil-borne life cycle of the pathogen. To test this hypothesis we investigated possible microevolution at two natural anthrax foci from the 2004 outbreak. If valid, then genotypes of strains isolated from near the surface at these foci should be on a different evolutionary trajectory from those below residing in deeper-laying horizons close to the carcass. Thus, the genetic diversity of *B*. *anthracis* isolates was compared conducting Progressive Hierarchical Resolving Assays using Nucleic Acids (PHRANA) and next generation Whole Genome Sequencing (WGS). PHRANA was not discriminatory enough to resolve the fine genetic relationships between the isolates. Conversely, WGS of nine isolates from near-surface and nine from near-carcass revealed five isolate specific SNPs, four of which were found only in different near-surface isolates. In support of our hypothesis, one surface-isolate lacked plasmid pXO1 and also harbored one of the unique SNPs. Taken together, our results suggest a limited soil-borne life cycle of *B*. *anthracis*.

## Introduction

Anthrax is a non-contagious infectious disease that principally afflicts domestic and wild ruminants but other animals including horses, donkeys, pigs, as well as humans are also susceptible to infection. The disease is characterized by a rapid lympho-haematogenous spread and production of toxins leading to internal hemorrhage and bleedings from orifices and death [1]. Today, anthrax is very infrequent and rare in most European countries [1], while it still poses a significant challenge in Albania [2], and in many other regions of the world such as Sub-Saharan Africa [3] and in parts of Asia [4]. While there is a general decrease in the number of anthrax outbreaks worldwide, anthrax is considered an extensively under-diagnosed and under-reported disease [5]. *Bacillus anthracis*, the etiological agent of anthrax, is a member of the *Bacillus cereus sensu lato* group [6] which comprises endospore-forming soil bacteria. Endospores are able to survive in extreme and unfavorable environmental conditions and remain viable in the soil over a long period of time. Due to the virulence of *B*. *anthracis* and because endospores are very stable in the environment and easy to cultivate, the bacterium is considered one of the most notorious agents to be potentially misused as a biological weapon or tool of bioterrorism [7,8].

Besides low interspecies diversity of the *B*. *cereus sensu lato* group on the genomic level, *B*. *anthracis* itself can be considered almost as a clonal organism with little, if at all, horizontal gene-transfer. Evolution is considered to be restricted to the limited reproduction phases of 20–40 generations during host infection while the ensuing endospores may lay dormant for years away from any host [9]. Consequently since its rather recent radiation into its three major branches A, B and C between 26 to 13 millennia ago [10] only a relatively small degree of genetic variations can be observed when comparing strains from different parts of the world [10,11]. Analysis of genomic data revealed variations mainly in Variable Number of Tandem Repeats (VNTR), Single Nucleotide Repeats (SNR) and Single Nucleotide Polymorphisms (SNP). A subset of the SNPs are representative for the three major lineages A, B and C of *B*. *anthracis* which can be further divided into thirteen distinct original canonical SNP (canSNP) sub-branches that robustly reflect the global phylogenetic relationships among *B*. *anthracis* strains [10,11]. Within a single distinct canSNP group, Multi Locus VNTR Analysis (MLVA) can be used to further differentiate strains, e.g., in outbreak investigations because of the higher diversity within these markers due to higher mutation rates in VNTRs compared to point mutations [9,10,12]. Finally, analysis of highly mutable SNR markers serves as a tool to discriminate between very closely related isolates even within a single MLVA genotype during enzootics [9,13]. This hierarchical fingerprinting system called PHRANA (Progressive Hierarchical Resolving Assays using Nucleic Acids) is commonly used for epidemiological investigation of outbreaks as well as for trace back analyzes in bioforensics [9,14] and for instance was used in the 2001 “Amerithrax” event [15].

From ingestion of *B*. *anthracis* endospores by grazing or via inhaling dust-particles various animal species can be infected [16]. Due to spilling of highly contaminated body fluids with or without dissection of cadavers by scavengers [17], massive dissemination of *B*. *anthracis* may occur in the surrounding soil constituting the natural reservoir of *B*. *anthracis*. These sites of spore contamination pose a high risk of reinfection for grazing animals [16]. However, outbreaks occur only sporadically with time intervals often ranging from years to decades and typically these outbreaks are seasonal following a period of hot-dry weather and rain fall [1].

In Italy, where anthrax is still enzootic, the disease is sporadic and it occurs, with few exceptions, in the central and southern regions, and on the major islands. Infections of humans are rare; mostly animals at pasture are affected [18,19]. In Italy, anthrax tends to occur where infected animals have been buried in the past. In the region of Basilicata in southern Italy, anthrax outbreaks are typically isolated, self-containing, and involve unvaccinated herbivores. During the summer of 2004, as a result of favorable weather conditions at “Pollino National Park”, a larger anthrax epizootic occurred. The affected area comprised 13 settlements and involved animals from 41 farms over an area of about 900 km^2^, with a livestock population of about 7,000 cattle and 33,000 sheep or goats. Within 40 days, 81 cattle, 15 sheep, nine goats, eleven horses and eight red deer succumbed to the disease [18]. Several animal carcasses were buried on high-altitude pastures (S1 Fig). From these infective bodies massive amounts of *B*. *anthracis* endospores were released into the surrounding soil, contaminating the surface above the burial site (Fasanella, unpublished results) probably due to diffusion into puddles of standing waters after heavy rainfall [16].

Despite the prevailing theory that in nature *B*. *anthracis* is an obligate pathogen restricted to a metabolically inactive endospore state outside the host, an early hypothesis [20] postulated multiplication of *B*. *anthracis* in “incubator areas” (soils rich in organic matter and calcium with a pH above 6.0 and an ambient temperature above 15.5°C). The sporadic occurrence of anthrax outbreaks under certain climatic and environmental circumstances could then be explained by local accumulations of *B*. *anthracis* that reach the required dose for infection of grazing animals due to multiplication in near-surface soil. A competing hypothesis suggests that these local accumulations emerge from the physical pooling of endospores in rainwater depressions because of the endospores’ hydrophobic surface character [16,21,22]. Furthermore, vegetative cells of *B*. *anthracis* were suggested to be unable to successfully compete with resident soil microbiota [23] and have never been found in natural environments. Also the clonal genetic character of the organism argues against frequent episodes of soil proliferation. Thus, the lifestyle of *B*. *anthracis* in the environment has jokingly been summarized as “sporulate or die” [1].

An increasing amount of laboratory findings, however, contrast with this established view of an obligate pathogen. Since other members of the genetically homogenous *B*. *cereus s*. *l*. group were found to multiply in guts of soil associated invertebrates [24], as a saprophyte in the rhizosphere of plants [25] and also in sterilized soil [26], it would be surprising if multiplication of *B*. *anthracis* is strictly limited to an animal host body. Indeed, under laboratory conditions multiplication was observed in the rhizosphere of grass plants [27] and recently the proliferation of *B*. *anthracis* within soil-dwelling amoebae was also confirmed [28]. Further, germination, multiplication and sporulation of *B*. *anthracis* have been observed in sterilized soil [29]. Additional experiments with bacteriophages showed that lysogeny of *B*. *anthracis* may enable the bacterium to colonize the intestinal tract of earthworms [30]. These findings suggest that *B*. *anthracis* is principally competent to grow in suitable soil environments, but this has not been directly observed in the environment thus far. Frequent probing of soils contaminated with *B*. *anthracis* over several years revealed isolates lacking one or both virulence plasmids [31,32,33,34]. This loss, on the background of the aforementioned laboratory findings, can be explained most simply by postulating a soil-borne life cycle of *B*. *anthracis*.

**Fig 1:**
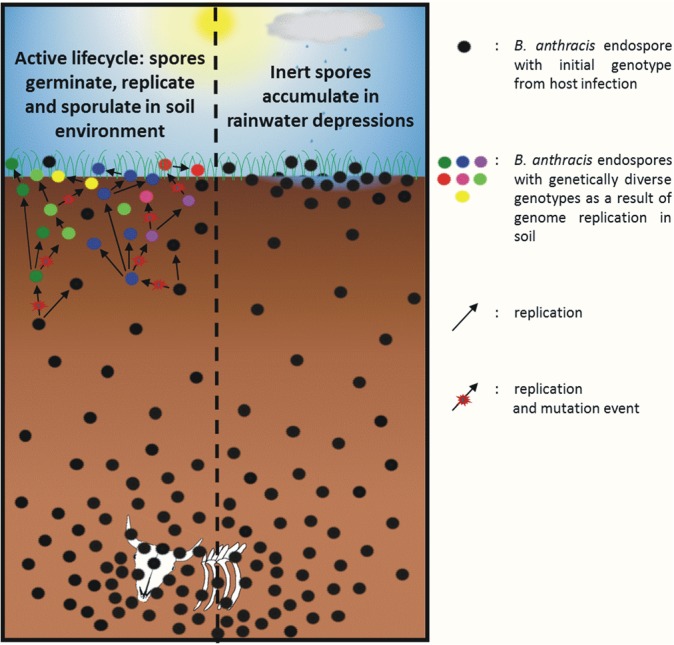
Hypothesis on an active life cycle of *B*. *anthracis* in soil environments. Soil surrounding the carcass at the lower part of the figure harbors a very high spore burden. The left part of the panel predicts massive soil proliferation of *B*. *anthracis* (hypothesis). In this case of an active near-surface life cycle, local accumulation of spores is suggested to be due to repeated rounds of germination, replication and sporulation in the near-surface soil environment. During genome amplification random mutations occur resulting in derived genotypes compared to the genotypes of the initial spore population within the carcass. The right part of the panel depicts events if there was no soil-borne life cycle of the pathogen (competing hypothesis). Inert spores are supposed to accumulate in rainwater depressions. Genotypes differing from the original animal-infecting population cannot be observed in near-surface isolates.

Bacteria and protists, such as the potential soil-dwelling amoeba-hosts for *B*. *anthracis* [28], are more abundant within the upper 25 cm of soil horizons compared to deeper layers of 100–200 cm probably due to higher availability of nutrients and bioavailable carbon mainly present in the top soil in grasslands, which constitute highly dense organic networks [35]. We would thus expect the potential proliferation of *B*. *anthracis* to occur rather in near-surface soil than in deeper layers. Regarding the “Pollino” burial sites, proliferation in the upper soil layers would then probably lead to accumulation of mutations and microevolution in the near-surface subpopulation of *B*. *anthracis* (Fig 1).

To test the hypothesis of a soil-borne lifecycle of *B*. *anthracis*, analysis of the genetic diversity among strains isolated from soil samples taken near the carcass (100–200 cm depth) and of near-surface derived isolates (5 cm depth) were conducted at three documented (Fasanella, unpublished data) anthrax carcass burial sites within Pollino National Park.

The hierarchical fingerprinting tool PHRANA (comprising canSNP analysis, MLVA and SNR analysis) was applied in this study to obtain insights into the diversity of *B*. *anthracis* in soil isolates from anthrax-foci. In order to obtain a high-resolution genetic fingerprint of the isolates, necessary for testing the hypothesis, whole genome sequencing (WGS) was conducted on nine isolates from near the surface and nine from close to the carcasses, respectively. The subsequent SNP discovery within this data-set of isolates was anticipated to be the tool with the highest resolving power for diversity investigation in a clonal organism such as *B*. *anthracis*.

## Material and Methods

### Collection of soil samples

**Fig 2:**
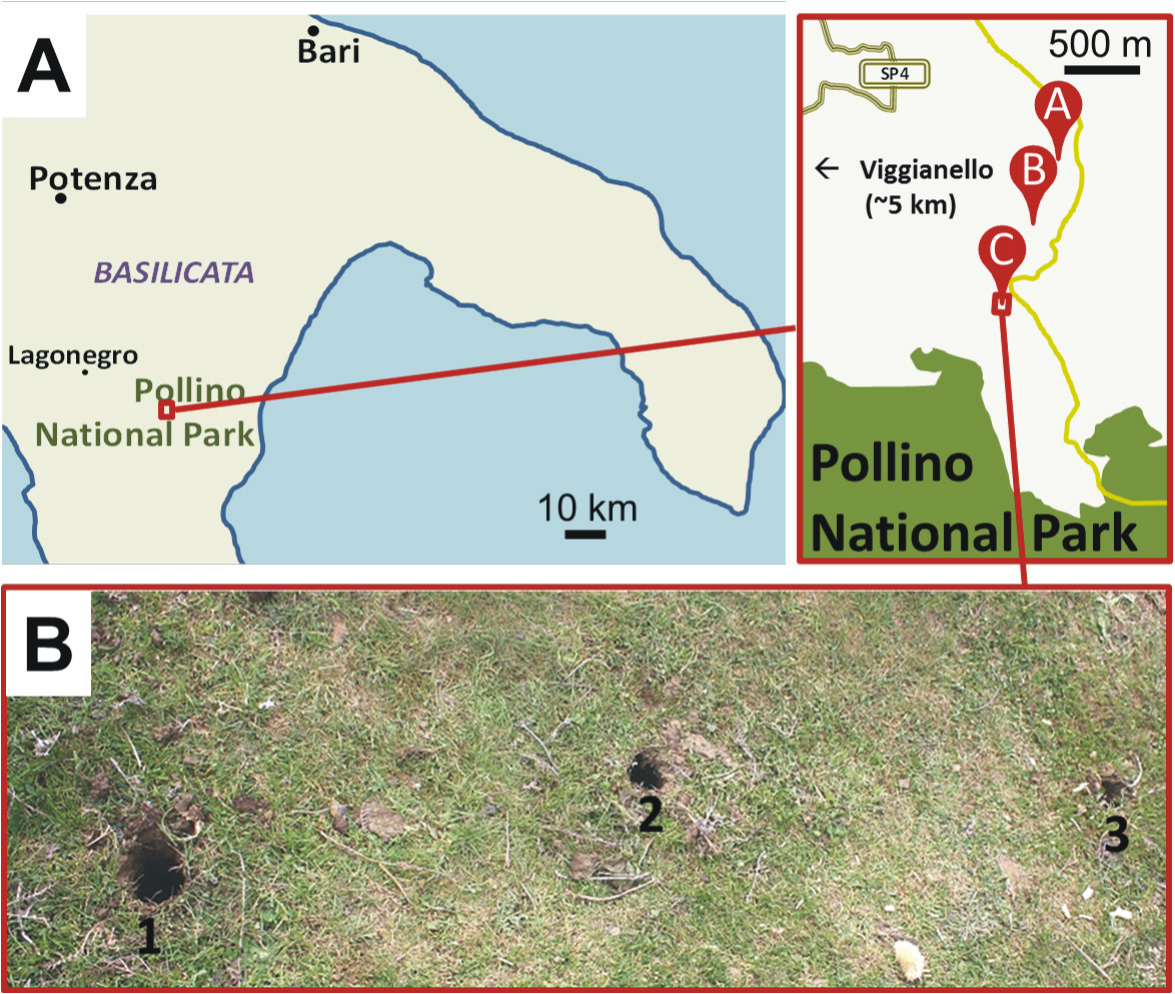
Location and details of sampling sites in southern Italy. Panel A indicates the locations of burial sites in Southern Italy (left) and positions of burial sites A, B and C at Pollino National Park (right). Panel B shows burial site C after sampling at positions 1, 2 and 3 (holes, approx. 50 cm apart).

Soil samples for this study were collected in May 2014 at Pollino National Park situated in the Basilicata region in the South of Italy, approximately 5 km east of the village of Viggianello (Fig 2A) with permission from the Italian authorities (Prevention Department of the *Azienda Sanitaria Potenza*) and the owner of the pasture land. Three different sites A, B and C were chosen. Three individual spots were selected for sampling from each of the three burial sites (Fig 2B, and Table 1). Soil samples were taken with a soil auger from 5, 100 and 200 cm below the surface. To avoid contamination of the deep soil samples with near-surface soil while pulling the drill out of the hole, all the soil coating the drilling core was removed with a clean knife and only the innermost part of the core was collected in a 50 ml sterile plastic tube.

**Table 1:** Strains of *B*. *anthracis* isolated from soil samples of Pollino National Park. Isolates characterized in Germany or Italy are labeled with numbers or letters, respectively. Isolate nomenclature is defined by burial site (letter) sampled position (number/), its depth (cm), and a successive number^1^ or letter^2^ throughout the manuscript, e.g., C1/5 cm– 1.

Burial site	Position	Depth (cm)	Isolates*
**A**	**1**	5	1, 2, 3, 4, 5, 6, 7, 8
		100	1
		200	1, 2, 3
	**2**	5	1, 2, 3, 4, 5, 6
		100	1, 2, 3, 4
		200	1
	**3**	5	1, 3, 4, 5, 7, 8, 9, 10, 11, 12
		100	1, 2, 3, 4, 5, 6, 7, 8, 9, 10, 11
**C**	**1**	5	1, 2, 3, 4, 5, 6, 7, 8, 9, 10, 11, 12, 13, 14, 15, 16
		100	1, 2, 3, 4, 5, 6, 7, 8, 9, 10, 11, 12, 13, 14, 15, 16
	**2**	5	1, 2, 3, 4, 5, 6, 7
		100	1, 2, 3, 4, 5, 6
		200	1, 2, 3
	**3**	5	1, 2, 3, 4, 5, 6, 7, 8, 9, 10, 11, 12
		100	1, 2, 3, 4, 5, 6, 7, 8, 9, 10, 11, 12, 13, 14, 15

*no strains were recovered from site B and isolates from soil samples A3/200 cm, C1/200 cm and C3/200 cm were not characterized in this study.

### Isolation of *Bacillus anthracis* from soil samples and inactivation

The Ground Anthrax *Bacillus* Refined Isolation (GABRI) was performed as described by [36]. After incubation at 37°C for 24 hours the suspected colonies appearing whitish and without hemolysis were picked and spread on Columbia blood agar. To reduce the chance for mutations during *in vitro* cultivation, bacteria were inactivated (for DNA extraction) after a single passage on solid growth medium. In rare cases of impurities another incubation step on selective agar was repeated. A few colonies were scraped off the plate and transferred into a 1.5 ml reaction tube filled with 500 μl 2% (v/v) Terralin PAA (Schülke & Mayr). Colony clumps were dissolved by rotating the inoculation loop or by pipetting. The reaction tube was filled up completely with 2% (v/v) Terralin PAA and incubated for 30 min for a proper inactivation of *B*. *anthracis* endospores which are vulnerable to peracetic acid [37], the active agent of Terralin PAA. Following centrifugation at 6000 x g for 2 minutes the supernatant was removed and the pellet resuspended in 1 ml phosphate-buffered saline (PBS). After two more washing steps with 1 ml PBS the pellet was stored at -20°C until further use. Work involving live *B*. *anthracis* was performed in a biosafety level 3 laboratory within a class III safety cabinet (glove box).

For each soil sample up to three aliquots were processed and from these ≥3 isolates (if present) were further processed in order to obtain a representative subpopulation. Names of single isolates comprise sampled position, their depths, and a running number. C1/5 cm– 1, for instance, indicates the first isolate from position 1 of burial site C in 5 cm depth. Strains which were further characterized are listed in Table 1.

### Extraction of DNA from inactivated culture material

For the isolation of DNA from inactivated bacteria suspensions, DNeasy Blood and Tissue Kit (Qiagen) was used as described in the manual for Gram-positive bacteria with the following changes. After cell wall lysis with lysozyme, 1 μl of RNase A (Qiagen) was added to each reaction tube and the mix was incubated at 37°C for another 10 min. Time for lysis with Proteinase K was doubled to 60 min and elution was done twice (150 μl and 100 μl, pooled) using sterile nuclease free Milli-Q water instead of Buffer AE. The DNA concentration of each eluate was quantified using the Qubit dsDNA HS Assay Kit (Life Technologies) according to the supplier’s protocol. DNA solutions were stored at -20°C until further use.

### Diagnostic real-time PCR for chromosomal and plasmid markers of *B*. *anthracis*

Real-time PCR for detection of *capC* (pXO2) and *pagA* (pXO1) were performed according to [1]. *Dhp61* (BA_5345) real time PCR on a specific chromosomal marker was used for identification of *B*. *anthracis* as described by [38]. Assays were conducted using either LightCycler 1.5, LightCycler 480 II (Roche) or the Stratagene MX 3000P (Agilent Technologies). Data analysis was performed with the respective associated software.

### Analysis of canonical Single Nucleotide Polymorphisms (canSNPs) by Melt Mismatch Amplification Mutation Assay (Melt-MAMA)

For canSNP typing of the *B*. *anthracis*-isolates Mismatch Amplification Mutation Assays (Melt-MAMA) were performed [39]. In short, for every canSNP assay two previously genotyped *B*. *anthracis* isolates were used as controls carrying either the ancestral or derived allele, respectively. The 20 μl reactions contained 1x LightCycler FastStart DNA Master SYBR Green-I, 4 mM MgCl_2_, about 5 ng template DNA, and primer mixtures specific for each assay. Real-time PCR was conducted using LightCycler 1.5 (Roche). Thermal profiles used for amplification and subsequent melting curve analysis were as published previously [39].

### Multi locus VNTR (variable number of tandem repeats) analysis (MLVA)

MLVA is a standard tool for *B*. *anthracis* genotyping [10]. Since most of the long and highly-repetitive VNTRs cannot not be determined by whole genome sequencing, lengths of amplified repetitive regions were analyzed by DNA-fragment-length analysis supported in part by Sanger sequencing, allowing for the calculation of repeat numbers or definite base-sequence, respectively. Fragment lengths of fluorescently labeled amplicons covering the whole VNTR locus were measured by capillary gel electrophoresis. In order to amplify the 31 different VNTR loci five conventional multiplex PCRs were carried out as described previously [40,41,42] with minor changes in primer concentrations.

PCR products were diluted 1:10 in water. A volume of 2 μl of each dilution and 0.5 μl of size standard Genescan 1200 LIZ (Applied Biosystems) were added to 17.5 μl Hi-Di Formamide (Applied Biosystems) and incubated at 95°C for 5 min to denature DNA. Fragment lengths were analyzed on the 3130 Genetic Analyzer (Applied Biosystems) and the associated GeneMapper software.

### Single nucleotide repeat (SNR) determination by fragment length analysis

Because of intrinsic technical-limitations of Ion Torrent technology the results of whole genome sequencing cannot be used for analysis of the highly mutable long homopolymeric regions. Therefore, fragment length analysis was employed to determine SNR lengths [13] (Table 2). Each of the four SNR-loci (HM1, 2, 6 or 13) were analyzed in single- or multi-plex PCR reactions. PCR products were diluted, denatured and run on the 3130 Genetic Analyzer (Applied Biosystems) with size standard Genescan 1200 LIZ (Applied Biosystems) using the associated GeneMapper software.

**Table 2:** SNR loci of *B*. *anthracis* analyzed. Information according to [43]. Names of loci in brackets are alternative names [44] of the same loci.

Locus name	location	GenBank Accession-#	5’–Position^1^
HM1 (CL33)	pXO2	NC_007323	60318
HM2-2* (CL10)	chromosome	NC_007530	4569999
HM6 (CL12)	chromosome	NC_007530	1448179
HM13 (CL35)	pXO2	NC_007323	34423

*: The marker is abbreviated as HM2 throughout this work.

^1^in the Ames Ancestor reference genome

### Ion Torrent next generation whole genome sequencing

DNA-library preparation for whole genome sequencing with the Personal Genome Machine (PGM, Life Technologies) required 100–1000 ng of high quality DNA in 50 μl nuclease free water. Since the DNA concentrations of eluates from genomic extractions were low because of low DNA yield and high elution volumes, DNA solutions were concentrated to 2–20 ng/μl. For this either the Concentrator Plus (Eppendorf) was used to evaporate aqueous solutions until the desired volume was reached or for DNA concentrations below 0.5 ng/μl several eluates from parallel genomic DNA extractions of a single isolate were pooled and precipitated with potassium acetate and ethanol. In the latter case, the pellet was washed twice with 75% ethanol to remove salt-contaminations, shortly dried at room temperature and finally dissolved in the desired volume of nuclease free water.

Whole genome sequencing of DNA of *B*. *anthracis*-isolates from Pollino National Park was carried out with the Ion PGM (Life Technologies). For this, library preparation was performed according to the Ion Xpress Plus Fragment Library Preparation Guide (Life Technologies) using a sonication method involving the Bioruptor system UCD-200 (Life Technologies) and subsequent end-repair (Life Technologies). The following settings of the Bioruptor were used to obtain fragment sizes of 400 bp: Time On/Off: 3x 15 min, 0.5 min/μM, min, Power level L (low). The 2100 Bioanalyzer (Agilent Technologies) was used for quality control after end-repair, size selection and adapter ligation following the instructions of Agilent High Sensitivity DNA Quick Start Guide (Agilent Technologies). DNA concentrations of the libraries were measured using the Qubit dsDNA HS Assay Kit (Life Technologies) and the DNA was diluted to 44.2 pM. Emulsion PCR and enrichment of template positive Ion Sphere Particles were performed according to the Ion PGM Template OT2 400 Kit (Life Technologies). Chip loading was carried out according to the manual using Ion 318 Chip Kit v2 (Life Technologies) in combination with Ion PGM Sequencing 400 Kit-chemistry (Life Technologies).

### Analysis of whole genome sequencing data–SNP calling

Sequencing output files were mapped to the genome *B*. *anthracis* Ames ancestor (GenBank entries: chromosome: AE017334.2; pXO1 AE017336.2; pXO2: AE017335.3) using bowtie2 [45]. A Variant Call File (VCF) was generated by SAMtools [46] for every isolate containing all variations compared to the reference genome including quality and coverage information. A python script was used to identify all single base variations that were both present in more than 75% of the reads and marked as high-quality bases. This analysis was carried out two times, once with a minimum coverage depth for variations of 15 and once with a minimum of 3. In order to avoid false negative as well as false positive results, the output data of the two approaches were compared. All results that indicated a SNP in one isolate compared to the others were re-checked by manual inspecting the respective BAM files using Tablet software [47]. Raw read data derived from genome-sequencing of the 18 analyzed Pollino isolates were deposited at the European Nucleotide Archive (ENA) under the sample accession numbers ERS723747 to ERS723764. The draft genome of one isolate, A3/5 cm– 1, (#ERS723747) has been published recently as “Pollino” reference strain [48].

### Determination of SNP distribution among strains isolated from anthrax loci via PCR with high resolution melting curve analysis (HRM-SNP)

In order to validate characteristic SNPs identified by whole genome sequencing in Pollino isolates and to determine the distribution of these SNPs in the other isolates, high-resolution melt (HRM) PCR assays were designed surrounding the SNP regions. Primer oligonucleotides were designed with the Primer-BLAST tool of NCBI [49] using the *B*. *anthracis* Ames ancestor genome (GenBank: AE017334) as a reference. Each primer pair was used in a 20 μl single-plex reaction (Table 3). For this, 0.2 μM of each primer pair (HRM SNP1-6 F + R), 3 mM MgCl_2_ and approximately 20 ng of template DNA were added to 1 × LightCycler 480 High Resolution Melting Master (Roche). Amplification and melting curve analysis was carried out on the LightCycler 480 II (Roche) using the following thermal profile for amplification: 95°C for 10 min, 35 cycles at 95°C for 10 s, 55°C for 10 s, 72°C for 10s. For the HRM-SNP 1 assay a touchdown PCR with annealing temperatures from 62°C to 57°C (step size 0.2) was performed to reduce unspecific PCR by-products. The following thermal profile was used for melting curve analysis: 95°C for 1 min, 60°C for 1 min and a final heating step to 95°C with a ramp rate of 0.02°C/s and continuous quantification at 530 nm.

**Table 3:** Oligonucleotides used for high resolution melting analysis of Pollino-SNPs.

Name	Sequence (5’– 3’)	SNP-position^1^
HRM SNP 1 F	CgTTTCATTTTAgCTgCggTA	1330849
HRM SNP 1 R	gTACgCTAATTTCCgTgCT	
HRM SNP 2 F	CgCgTTTTACAACCAAAAggT	2286596
HRM SNP 2 R	AAACTCAgCTAATTCACTCgCAT	
HRM SNP 3 F	TTTCgTATTTCgCTACATCTTTTCCA	3240167
HRM SNP 3 R	AgCgTTACTTCAgTTAAAgCCA	
HRM SNP 4 F	CCTTTACgAATTgCTggTgC	3881699
HRM SNP 4 R	TCAgTAgCTggAgTAgATCCAA	
HRM SNP 5 F	CgATACgTAAATCTCCCTCTTCCA	4059291
HRM SNP 5 R	ggTggCACgATTTTACTTTCgTA	
HRM SNP 6 F	ATggCTTgTgTAAgCgTgAgA	4380690
HRM SNP 6 R	gCTgCAAAgCgTTTCAAAAAgA	

^1^with reference to *B*. *anthracis* strain Ames Ancestor

### Sanger sequencing

To verify results from SNR- and SNP-typing Sanger sequencing was conducted. For this, conventional PCR was carried out to generate templates for the chain-termination sequencing-PCR. PCR products were purified using the QIAquick PCR Purification Kit (Qiagen) according to the manufacturer’s protocol and used as a template for the BigDye Terminator v3.1 Cycle Sequencing Kit (Applied Biosystems). Prior to sequencing by capillary gel electrophoresis the product of chain-termination PCR were purified using DyeEx 2.0 Spin Kit (Qiagen) and 3 μl of the eluate were mixed with 12 μl of HiDi Formamide (Applied Biosystems). The mix was applied to the 3130 Genetic Analyzer (Applied Biosystems) for sequencing and data analysis was carried out with the ABI Prism 3130 DNA Analyzer software (Applied Biosystems).

## Results

### Initial classification and canSNP-typing of *B*. *anthracis* isolates from Pollino National Park assign strains to canSNP group A. Br. 011/009

All isolates retrieved by the GABRI method [36] were identified as *B*. *anthracis* by diagnostic real time PCR on chromosomal markers *dhp61*, *pagA* (pXO1) and *capC* (pXO2) (data not shown). Notably, one isolate C3/5 cm– 1 was found to lack virulence plasmid pXO1. No *B*. *anthracis* could be isolated from soil samples of site B. At the time of sampling this burial site was flooded because of rainfall in the preceding weeks. Nevertheless, samples were collected from both soil and water. All of these turned out to be negative for *B*. *anthracis* (data not shown). However, in the previous year (2013) this site has been successfully sampled for *B*. *anthracis* (Grass and Fasanella, unpublished results) indicating a negative effect under such conditions of flooding on recovery or viability of *B*. *anthracis*.

The classification of *B*. *anthracis* into 13 canonical SNP-groups within the three major branches A, B and C is used for subtyping of strains on a global phylogenetic background [10]. Because all Pollino soil isolates were associated with one outbreak, all strains should have the same canSNP-type and therefore the method cannot be considered appropriate for analysis of genetic diversity. However, canSNPs were used as a preliminary test to eliminate the possibilities of isolates being the result of a laboratory contamination or that the diseased cow had been infected by more than one canSNP-genotype of *B*. *anthracis*. As expected all isolates belonged to the A. Br. 011/009 canSNP group (data not shown) as defined by [11] as a subset of the initial canSNP-groups [10] indicating that all isolates originated from the collected soil samples. The lack of virulence plasmid pXO1 in isolate C3/5 cm– 1 was confirmed by a second PCR for a different marker (PS-1 from pXO1).

### MLVA lacks sufficient discriminatory power for analysis of genetic diversity in Pollino isolates

Multiple Loci Variable Number of Tandem Repeat (VNTR) analysis (MLVA) is commonly used in a series of progressive hierarchical resolving assays for subtyping *B*. *anthracis* and has a higher resolution power than canSNP analysis [9]. Because of the variability in the repetitive VNTR regions makes it possible to obtain broader information about the genetic diversity within a certain population. Therefore, 114 randomly chosen soil isolates were screened for variations in their 31 MLVA markers (Table 4). All isolates were assigned to the MLVA cluster A1.a [41]. Only three fragment length variations were observed in a total of 3534 single markers, that is 31 markers for each of 114 isolates. Strains with length deviations compared to the major allele in the investigated population were: C1/100 cm– 1; A2/200 cm– 1 and A3/100 cm– 9. As a randomly picked control, Sanger sequencing verified the variance in Bams15-marker of isolate C1/100 cm– 1. The loss of virulence plasmid pXO1 in isolate C3/5 cm– 1 was also verified by MLVA since no fragment for VNTR pXO1 was detected (Table 4).

**Table 4:** Multiple 31-Loci VNTR of *B*. *anthracis* isolates from Pollino. The predominant alleles of fragment length analysis for 31 markers of MLVA in 114 randomly chosen isolates are shown. Variations in these markers found in some isolates are shown in boldface (32 markers are shown since VNTR32 and bams1 represent the same VNTR locus).

MLVA marker	Fragment length (bp)repeat <#>	MLVA marker	Fragment length (bp)repeat <#>	MLVA marker	Fragment length (bp)repeat <#>
bams1	425 <13>	bams30	885 <73>	vrrC2	594 <19>
bams3	608 <30>	bams31	774 <64>	pX01	121 <7>, **124 <8>**, **-** ^1^
bams5	378 <7>	bams34	429 <7>	pX02	133 <7>
bams13	452 <29>	bams44	420 <7>	VNTR12	113 <6>
bams15	612 <46>, **576 <42>**	bams51	498 <9>	VNTR16	269 <8>
bams21	660 <9>	bams53	234 <8>	VNTR17	386 <4>
bams22	711 <13>	CG3	148 <1>	VNTR19-2	93 <5>
bams23	633 <9>	vrrA	307 <4>	VNTR23	192 <4>
bams24	599 <9>	vrrB1	224 <17>	VNTR35	106 <3>
bams25	394 <4>	vrrB2	158 <14>, **149 <13>**	VNTR32	502 <12>
bams28	498 <14>	vrrC1	603		

^1^Because of loss of virulence plasmid pXO1, one strain C3/5 cm– 1 yielded no fragment for VNTR marker pXO1.

The results of MLVA indicated that for genetic diversity investigation within a population of very closely related strains originating from Pollino burial sites, the mutability of these VNTRs was not sufficiently high and thus, the method was not discriminatory enough. However, the observed few variations were found exclusively in near-carcass depths (100 cm and 200 cm). Therefore, MLVA failed to support our hypothesis of microevolution in soil of anthrax foci.

### SNR analysis reveals slightly higher genetic diversity in near-carcass than in near-surface isolates

The most variable VNTR-markers and thus, the regions with the greatest resolving power during outbreak events in terms of the genetic diversity in *B*. *anthracis* are Single Nucleotide Repeats (SNR) consisting of homopolymeric stretches of (mostly) A or T. Four of these loci are commonly investigated. Because they showed the highest variability in previous studies [13,19], these were also analyzed in this project. Several *B*. *anthracis* strains from our collections were used as standards since their SNR lengths were known (data not shown).

SNR lengths were calculated by subtracting the respective bases of the primers and of flanking regions of the SNR-repeats from the fragment represented by the respective signals (peaks). Table 5 indicates the calculated SNR homopolymer lengths of the tested isolates. To ensure the investigated population being representative for the burial sites’ soil population, results of SNR analyses performed in Italy and Germany were combined. A total of 18 SNR length differences within the loci HM1 and HM2 were detected among 174 isolates from Pollino (Table 5). Not a single alternative allele was identified in HM6 or HM13 markers in all tested isolates (data not shown). A subset of strains exhibiting variations were re-examined by Sanger-sequencing to confirm these results. Thus, fragment length analysis combined with Sanger sequencing yielded reproducible SNR results. According to their SNR profiles, each isolate was assigned to a SNR-specific subgenotype (SGT1-6, Table 5). The majority (156 isolates) belonged to SGT1. For the other SNR-variants (SGT2-6) a ratio of 10 (near-surfaces) to 8 (near-carcass) was observed in 101 (near-surfaces) to 73 (near-carcass) isolates tested. This represented approximately a very similar percentage of occurrences of SNR-variants for near-surface (9.9%) and near-carcass (10.9%). The distribution of the variant SGTs in soil seemed to be random, especially in case of SGT3 and SGT4, since they were found in both burial sites (Table 5). Further, the low penetrance of most of the SGTs among the investigated isolates excluded clear statements about the genetic diversity, which seemed not to be higher among near-surface isolates of our tested population. However, the most abundant variant SGT, SGT2, was found in a slightly higher ratio in the near-surface soil of site C (6 of 64 isolates from 5 cm depth = 9.4%) compared to near-carcass isolates (3 of 43 isolates from 100–200 cm depth = 7.0%). Additionally, the only SNR length variation with a greater deviation than one bp compared to the major genotype SGT1 was found in the near-surface of site C with only one observed isolate (SGT6, -4 bp in HM1 compared to SGT1).

**Table 5:** SNR sub-genotypes according to length differences in markers HM1 and HM2.

Subgenotype	SNR length (bp) HM1:HM2	Number of isolates	Isolation site: depth (ratio of # of isolates with variant SGT of respective site/# of total isolates from depth X of the respective site)
SGT1	19:21	156	Major genotype
SGT2	**18**:21	9	Site C: 5 cm (6/64 = 9.4%), 100–200 cm (3/43 = 7.0%),
SGT3	**20**:21	3	Site C: 100–200 cm (1/43 = 2.3%) Site A: 5 cm (2/37 = 5.4%),
SGT4	19:**20**	2	Site A: 100–200 cm (1/30 = 3.3%), Site C: 100–200 cm (1/43 = 2.3%)
SGT5	19:**22**	3	Site C: 5 cm (1/64 = 1.6%), 100–200 cm (2/43 = 4.7%)
SGT6	19:**17**	1	Site C: 5 cm (1/64 = 1.6%)

### Genome sequencing revealed six SNPs in 18 Pollino isolates

Due to the genetically monomorphic character of *B*. *anthracis*, SNP analysis by whole genome sequencing has been served as the tool with the highest resolving power for investigation of genetic diversity within a single outbreak population [50] similar to the situation in Pollino soil isolates. Thus, microevolution caused by a predicted soil-borne life cycle of *B*. *anthracis* may also be tested comparing the SNP distribution between strains taken from near the carcass and those of near the surface. To investigate this, three isolates from both 100 cm and 5 cm each from three different sampling sites A3, C1 and C3 (18 isolates in total) were genome sequenced. Strains were chosen randomly being the first three strains isolated from each soil sample (A3, C1 and C3, both at 5 cm and 100 cm, respectively) that were confirmed as *B*. *anthracis* by diagnostic *dhp61* real-time PCR. The sequencing depth was on average 40-, 50- and 80-fold for the chromosome, pXO2 and pXO1, respectively.

Downstream analyses of the whole genome sequencing data revealed six SNP positions among the 18 isolates (Table 6). Additionally, 351 shared SNPs between Pollino isolates were detected that had the same allelic state in all sequenced isolates but these positions showed a different allelic state in the Ames ancestor reference genome. These also included the canSNPs separating Pollino isolates (A.Br. 011/009 cluster) from Ames ancestor (A.Br. Ames cluster).

**Table 6:** SNPs in Pollino isolates detected by whole genome sequencing. SNPs are numbered according to their position in the reference genome^1^ (*B*. *anthracis* Ames Ancestor; GenBank accession: AE017334). The bases at the respective positions are shown for Pollino isolates and the reference^2^. SNPs are highlighted in italics.

SNP number	1	2	3	4	5	6
Position (bp)^1^	1330849	2286596	3240167	3881699	4059291	4380690
Reference (base)^2^	C	A	G	G	G	A
A3/100 cm– 1	C	A	G	G	G	C
A3/100 cm– 2	C	A	G	G	G	A
A3/100 cm– 3	C	A	G	G	G	C
A3/5 cm– 1	C	A	G	G	G	A
A3/5 cm– 3	C	A	G	G	G	A
A3/5 cm– 5	C	A	*A*	G	G	A
C1/100 cm– 1	C	A	G	G	G	A
C1/100 cm– 2	C	A	G	G	G	A
C1/100 cm– 3	C	A	G	G	G	A
C1/5 cm– 1	C	A	G	G	G	A
C1/5 cm– 2	*T*	A	G	G	*A*	A
C1/5 cm– 3	C	A	G	G	G	A
C3/100 cm– 1	C	A	G	G	G	A
C3/100 cm– 2	C	A	G	G	G	A
C3/100 cm– 3	C	G	G	G	G	A
C3/5 cm– 1	C	A	G	*A*	G	A
C3/5 cm– 2	C	A	G	G	G	A
C3/5 cm– 3	C	A	G	G	G	A

Four of the six SNPs appeared in near-surface isolates. Isolate C1/5 cm– 2 even harbored two different SNPs (Table 6). Notably, SNP 4 was detected in isolate C3/5 cm– 1, which had been found to lack virulence plasmid pXO1. This loss was further confirmed by whole genome sequencing since coverage of pXO1 in C3/5 cm– 1 was negligible (data not shown). Five SNPs (1–5) were present in just one single isolate whereas SNP 6 was detected in A3/100 cm– 1 as well as in A3/100 cm– 3. Thus, this result suggested that this SNP had a higher prevalence at site A and that it might be possible that some of the non-sequenced isolates from site A also harbor SNP 6.

Next, we checked if the SNPs in the isolates have resulted in silent, missense or non-sense mutations in any of the bacterium’s genes or if intergenic regions were affected by these changes. For this, the respective regions or open reading frames were analyzed (Table 7). Nucleotide exchanges at SNPs 3 and 6 resulted in silent mutations, whereas those at SNPs 1, 2, 4 and 5 led to amino acid changes. For SNP 1, this caused an introduction of a positive charge, for SNP 2 the loss of a hydroxyl-side-chain, for SNP 4 the exchange of a more rigid and hydrophobic proline to more flexible, hydrophilic serine and for SNP 5 the change from a shorter, hydrophobic to a more bulky, hydrophobic side-chain in leucine or phenylalanine, respectively. While, the predicted phenotypic functional consequences of these mutations remain unknown, at least for SNPs 3, 5 and 6 functional alterations in the affected proteins can be expected to be minor (Table 7).

**Table 7:** Predicted functional consequences of SNP polymorphisms on the translational level. The SNP numbers (#), positions in the reference and the respective variant alleles for the SNP (Mut.) and the reference (Ref.) are shown as well as the potential amino acid (AA.) changes in the affected gene products and the gene designations.

#	Position	Ref.	Mut.	AA. exchange	Gene product	Gene
1	1330849	C	T	Gly 117 Arg	conserved hypothetical protein / thioreductase	GBAA_1404
2	2286596	A	G	Thr 160 Ala	O-methyltransferase family protein	GBAA_2456
3	3240167	G	A	-^1^	putative iron compound ABC transporter	BA_3531
4	3881699	G	A	Pro 291 Ser	acetyl-CoA acetyltransferase	GBAA_4240
5	4059291	G	A	Leu 135 Phe	*comG* operon protein 4	GBAA_4463
6	4380690	A	C	-^1^	ribosomal protein L35	GBAA_4818

^1^silent mutation

### HRM for analysis of SNP distribution in Pollino isolates

SNP discovery in the genome-sequenced Pollino isolates yielded a number of 6 point mutations that appeared to be specific for the population at the investigated burial sites. To investigate if these SNPs were more wide-spread even in other Pollino-isolates, the 96 remaining isolates which were not genome-sequenced (Table 1) were analyzed by interrogating for these SNPs. All observed mutations at the positions of SNPs 1–6 resulted in base pair exchanges from G + C to A + T or vice versa with SNPs 1–5 being transitions and SNP 6 a transversion. In order to investigate the distribution of SNPs discovered by whole genome sequencing among all non-sequenced isolates, assays for High Resolution Melting curve analysis (HRM) were designed targeting the six SNP positions (Table 6). These SNP assays were tested for all remaining isolates from the respective burial site where each SNP originated from (SNP 1, 2, 4 and 5 were tested in all isolates of burial site C; SNP 3 and 6 were tested in all isolates of burial site A).

**Fig 3:**
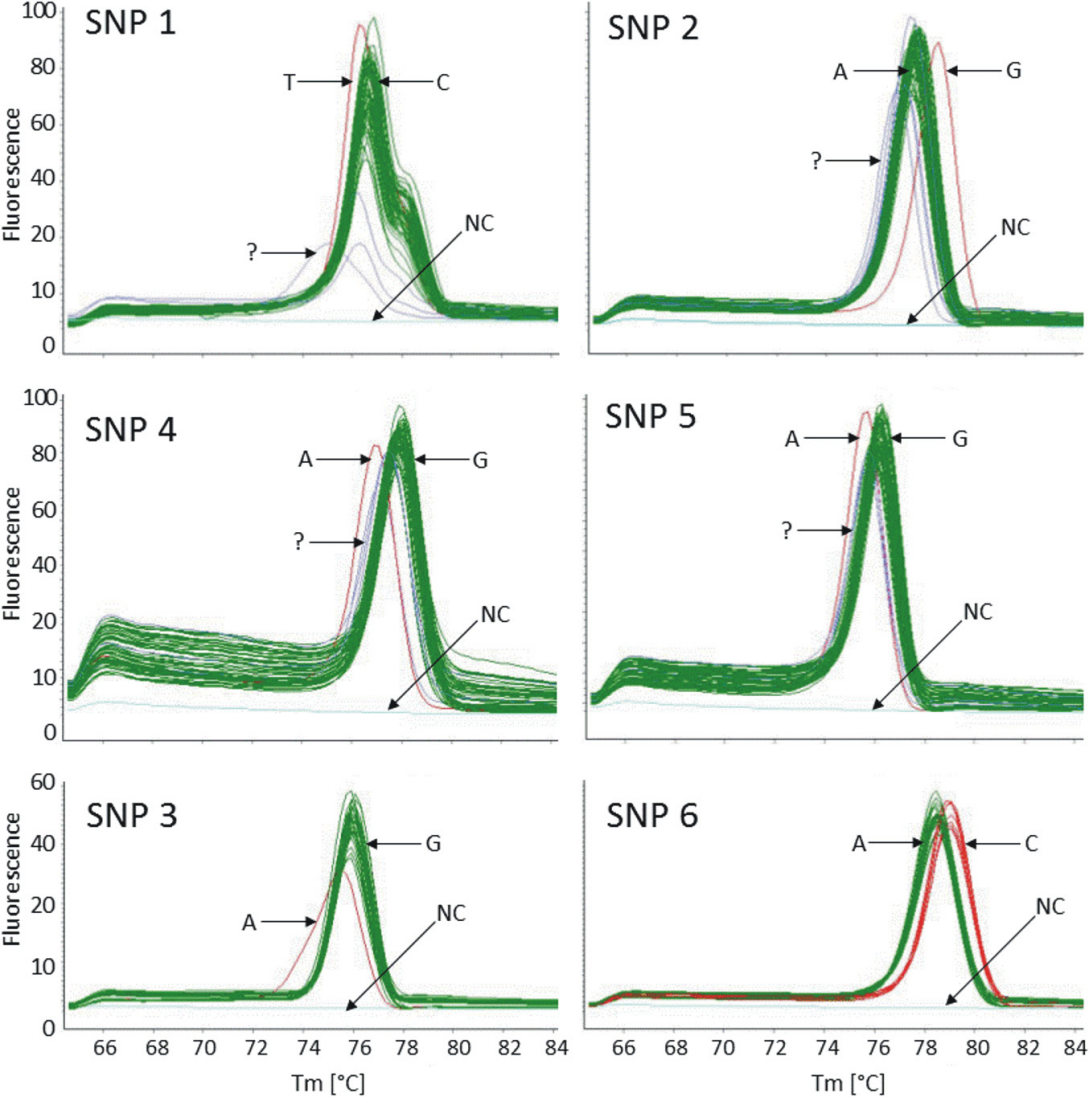
Temperature-dissociation (melt) curve derivatives of HRM-SNP. The relative fluorescence signal is plotted against the melting temperature (°C) for each SNP. Curves showing ancestral alleles (same base as predominant Pollino allele) are displayed in green. Curves indicating derived alleles (variant allele at the SNP-position) are displayed in red. The respective bases at the SNP position (“G” or “A”) are indicated for each allele group. Inconsistent curves (“?”) are displayed in blue. Negative controls (“NC”) are displayed in light-blue.

Melting curve analysis revealed melting temperature shifts (ΔTm ~ 0.5°C) of isolates harboring SNP-alleles unique for Pollino- (Fig 3: derived allele, red curves) compared to isolates with the respective other SNP-allele (Fig 3: ancestral allele, green curves). Amplicons that exhibited unexpected deviations in curve shape or melting temperature (Fig 3: blue curves) were sequenced by Sanger sequencing. None of these were found to contain a SNP, thus, all were ancestral. The allele-specific Tm values for the respective SNPs were on average as follows: SNP 1: ancestral allele (“C”): 76.9°C; derived allele (“T”): 76.5°C. SNP 2: ancestral allele (“A”): 76.7°C; derived allele (“G”): 78.4°C. SNP 3: ancestral allele (“G”): 76.0°C; derived allele (“A”): 75.4°C. SNP 4: ancestral allele (G): 77.9°C; derived allele (A): 77.0°C. SNP 5: ancestral allele (G): ~76.1°C; derived allele (“A”): 75.6°C. SNP 6: ancestral allele (“A”): ~78.5°C; derived allele (“C”): ~79.0°C. Taken together, SNP 1, 2, 3, 4 and 5 were identified as isolate-specific SNPs since they were not present in any other isolate. However, SNP 6, which was observed in A3/100 cm– 1 as well as in A3/100 cm– 3 (WGS), was also present in A3/100 cm– 5, A3/100 cm– 10, A2/100 cm– 1, A1/5 cm– 1 and A3/5 cm– 10 (Fig 3).

Overall reproducible results were obtained by analysis of different genomic levels from *B*. *anthracis* isolates exhibiting increasing resolution in genetic diversity analysis from canSNPs, to 31-loci MLVA, which lacked discriminatory power, to 4-loci SNRs and SNPs from whole genome sequencing. These new SNPs served as suitable markers for genetic variability interrogation of soil *B*. *anthracis*-population at Pollino burial sites. We could show that genetic diversity ratios between near-surface and near-carcass population were from 0 to 3 (near-surface allele variations/near-carcass allele variations) for MLVA, 10 to 8 for SNR analysis and 4 to 1 for unique Pollino-SNPs determined by whole genome sequencing.

## Discussion

### Loss of plasmid pXO1 in continuously present *B*. *anthracis* in Pollino soil hints on the possibility of microevolution

After the anthrax epidemic that occurred in Pollino National Park in 2004 several other outbreaks occurred in Basilicata region confirming that this region could be considered an area at high risk of anthrax in Italy and Europe. The last outbreak occurred in early October 2014. A cow became infected on a farm and after slaughtering of the animal a farmer developed skin lesions that were confirmed to be cutaneous anthrax. Consequently, the farmer was treated with antibiotics and remaining livestock was vaccinated (Fasanella, unpublished data). The genotype responsible of the outbreak was similar to the *B*. *anthracis* strains responsible of the outbreak that occurred in Basilicata region during the summer of 2011. The investigation on the origin of the outbreak indicated that the animal became ill by ingesting forage from the area in which the anthrax epidemic of 2011 had occurred (Fasanella, unpublished data).

The GABRI method [36], which was used in this project, turned out to be highly suitable for isolation of *B*. *anthracis* from soil samples of the Pollino burial sites. Altogether, strains were isolated from all tested soil samples except of those from burial site B, which was found flooded with rain water. Since *B*. *anthracis* could be isolated from this site previously (Fasanella and Grass, unpublished results) and on the background of the prevailing theory of *B*. *anthracis* accumulating in such rainwater depressions [16] we expected positive results in samples from this site. The failure of obtaining new isolates, however, apparently contradicts that notion.

Probing burial site C, isolate C3/5 cm– 1 exhibited a loss of virulence plasmid pXO1, while other isolates were found to harbor two copies of pXO1 per chromosome copy as indicated by the coverage ratio of 1:2:1 of chromosome, pXO1 and pXO2, respectively (data not shown). This confirms the recent findings from another strain of the same MLVA genotype, A1.a, which exhibited a very similar ratio of the three genetic elements [51]. Possibly, the loss of pXO1 in isolate C3/5 cm– 1 could be an artifact of growth during strain isolation from soil. However, such a loss in the laboratory was determined to be rare [52], and since DNA of the bacteria from Pollino analyzed in this study was extracted after a single passage, secondary loss during isolation can likely be ruled out. While isolation of *B*. *anthracis* lacking pXO2 or both virulence plasmids from contaminated soils is well known, natural pXO1-negative isolates are rather rare but emerged exclusively from soil sampling [31,32,34,52]. Such strains represent an evolutionary cul-de-sac as they are likely no longer able to successfully infect new hosts due to the lack of anthrax toxins [34]. Since pXO1-negative isolates were not detected in infected animals during the outbreak in 2004 (Fasanella, unpublished), it is possible that plasmid-loss occurred in the soil environment. The obvious assumption concerning the absence of pXO1 in isolate C3/5 cm– 1 is that as a consequence of a soil-borne life cycle the plasmid was spontaneously lost during replication. Thus, the finding of a pXO1-negative near-surface isolate provides a first support for our work hypothesis.

### The power of a hierarchical genotyping approach for investigating genetic diversity in outbreak strains

In this study the hierarchical fingerprinting system PHRANA which is commonly used in anthrax bioforensics and epidemiology [9], was applied for investigating the diversity of *B*. *anthracis* soil populations at Pollino burial sites. Because of low mutation rates, canSNPs are valuable only for broad phylogenetic analysis of different *B*. *anthracis* strains [12] and therefore provide insufficient resolution power for diversity investigation during outbreaks. Thus, canSNP analysis was conducted as a preliminary test to confirm the association of the characterized strains to the Pollino outbreak. All soil isolates were assigned to A. Br. 011/009, which is predominant in southern Italy [18].

MLVA typing provides greater resolving power than canSNP analysis because of the higher mutational rate in VNTRs suggested to be in the order of <10^−5^ to >10^−4^ mutations per locus and generation [9]. Besides the higher number of possible allelic states (multiple length variations for each locus) compared to SNPs (in general only two alternative alleles in any given population), elevated mutation capacity is also due to higher error rates during replication. Backward-slipping of DNA-polymerase may lead to duplication of a repeat unit and thus increase the number of tandem repeats. Conversely, decreases in the overall repeat count may accrue by skipping repeats via forward-slipping [53].

Despite its greater variability, the VNTR-based investigation of the 2004 outbreak revealed the same MLVA genotype A1.a in all 53 animal isolates tested [19]. Therefore, we expected to observe the same genotype in all soil isolates, with a chance of variations due to a soil-borne life cycle if existing. While the major genotype matched with previous results [19], three variations in fragment length were observed, each in a different MLVA-marker and strain, respectively. There are alternative models to explain the origin of these repeat count changes. Co-infection of an animal with more than one MLVA-genotype was recently described [54], but this possibility is negligible for the Pollino sampling sites since each MLVA-variant allele was found only in a single isolate among 114 tested. If variations were the result of soil replication, isolates must have undergone relocation from near-surface, where we hypothesize soil cycling to occur, to deeper layers where the carcass is located and possibly below that. This should be possible, because translocation with changing spatial vectors can be expected (*B*. *anthracis* was isolated from near-surface soil of the burial sites at 5 cm, away from the carcass at about 100 cm depth) and also the top down movement of spores is likely because of successful isolation of *B*. *anthracis* from a depth of 200 cm at both sampling sites (Table 1). However, it seems more plausible that exponential growth during the course of infection led to incorporation of these variations in the original diseased host animal. For *B*. *anthracis* such events have not been thoroughly investigated yet. However, Johansen and co-workers found different MLVA profiles of *Mycobacterium avium* subsp. *hominissuis* in a single pig and stated that mutations during infection could explain single-locus variations [55].

Therefore, the most probable explanation for these changes allelic states of MLVA markers found in soil isolates near the carcass is that these variations arose during late phases of host infection and thus, do neither support nor exclude the hypothesis of a soil-borne life cycle of *B*. *anthracis*. Anyway, MLVA provides insufficient resolution power since only three single variations were found in 3534 tested markers (testing 31 loci in 114 isolates).

Finer genetic resolution was observed by SNR analysis, which revealed five different (other than the predominant SGT1) SNR alleles among 696 tested markers (4 loci, 174 isolates). Similar to VNTRs, mutations in SNRs are most likely due to slipped-strand miss-pairing during replication but occur with higher frequencies of 6 x 10^−4^ per locus [9]. Especially locus HM1 on plasmid pXO2 (Table 5) is known to exhibit considerable allele variability with observed repeat lengths from 9 up to 60 nucleotides [13]. However, determination of the variable repeat lengths is challenging and several approaches have been tested to overcome these hurdles. Fragment length analysis [13,56] and Sanger- [40] or occasionally pyro-sequencing [57] are used. Unfortunately, no reliable information from SNR loci can be expected from whole genome sequencing data obtained by the PGM system and possibly other platforms since the Ion Torrent technology, which was used in this study, was reported to exhibit high error rates when sequencing homopolymeric stretches [58]. Thus, for interrogating the genetic diversity in SNR loci in *B*. *anthracis* isolates from Pollino the established approach of fragment length analysis followed by confirmation of selected variations by Sanger sequencing served as a medium-level time- and cost-effective but reliable methodical alternative. Both fragment length analysis and Sanger sequencing yielded identical results.

The major SNR genotype (SGT1, Table 5) found in 156 of the 174 tested isolates, was identical to that observed in the same geographic region from the original 2004 Pollino-outbreak investigation [19]. SGT2 and SGT3 as well as SGT5 were also observed earlier [19] but infected animals were found at distances of least 15 km from the now sampled burial sites. In contrast, SGT6 and SGT4 (Table 5) were not found during the 2004 enzootic outbreak. Interestingly, the frequency of detected SNR variations between the two cow burial sites A and C was found to be 3 to 15 (site A to site C) (number of screened isolates was 67 to 107, respectively) suggesting an evolutionary more active site C.

As for MLVA, three possibilities may be considered regarding the origin of these SNR variations. For those changes that appeared both in the 2004 enzootic outbreak and were now present in multiple soil isolates, the obvious explanation would be an infection by more than one genotype. Because of high homoplasy in these markers [9], a phylogenetic explanation on the origin of the observed variations can hardly be given and therefore these rather randomly distributed variations might have originated during the course of infection as well. This would agree with earlier findings [44], who compared SNR profiles of *B*. *anthracis* soil isolates distributed around carcass sites of bisons diseased from anthrax. The authors found similar distributions of SNR variations within soil samples of single carcass sites which were proposed earlier to be acquired during host passaging [43].

In total, there was no higher genetic diversity in SNR loci in near-surface soil compared to near-carcass layers at both burial sites (near-surface to near-carcass, site A: 2 to 1 differences in 37 to 30 isolates; site C: 8 to 7 differences in 64 to 43 isolates, respectively). Although the distribution of SGT2 showed a higher prevalence at 5 cm depth compared to deeper layers and the appearance of a near-surface isolate-specific 4-bp-shift in HM1, homoplasy in these markers and the low penetrance of variant SGTs impeded clear statements on the exact extend of genetic diversity. Thus, similar to MLVA, SNR data neither supported nor contradicted our hypothesis of a *B*. *anthracis* life cycle in near-surface soil.

### Distribution of unique SNPs as indicators of soil replication

Whole genome sequencing more and more becomes the tool of choice in molecular epidemiology of infectious diseases and was already used for investigation of anthrax outbreaks [50,59,60]. The Ion Torrent PGM platform was used for this project, which has been described as suitable for correct SNP calling [61]. The relatively high raw read-error rate of about 1.8% may be compensated for by sequencing coverage depths of 30–40 or more, which was achieved in this work. Limitations in detecting insertions and deletions (indels) and high error rates concerning homopolymeric regions were reported for the PGM [61]. Therefore and because MLVA and SNR analysis already gave reliable information about regions with high frequencies of indels, we limited the whole genome data analysis on SNP calling. This approach revealed six SNP positions in total when comparing nine randomly picked isolates each from near-surface and from near-carcass. While the possibility of false-positive SNPs may be negligible, there might be a certain chance for false-negative SNPs (*i*.*e*. SNPs that were not detected), both due to the conservative SNP calling quality filters of a minimum coverage depth of 15 with at least 75% of the reads containing the respective mutation.

Similar to variations obtained by MLVA and SNR, the observed SNPs might have been incorporated during laboratory culturing of strains after sampling. The *in vitro* mutation rate of *B*. *anthracis* single nucleotide variations, which is due to DNA-polymerase errors, was determined to be 8.3 × 10^−10^ mutations/bp/generation, about 10–20 times lower than the estimated rate for *in vivo* mutations [50]. All tested strains of this study have undergone only a single passage. In a Swedish anthrax outbreak the authors have not found any mutation in three different colonies after five passages [50]. Thus, the possibility of *in vitro* mutation being the source of the SNPs should be negligible. Instead, given the higher mutation rate *in vivo* than *in vitro*, SNPs might be incorporated during host passaging. This is possibly the source of SNP 6, which was found in several isolates from different depths, but exclusively at burial site/cow A. In case of SNP 6, it is also plausible that it was already present in the initial infectious load of cow A and thus, in this scenario, possibly a multiple-genotype infection had occurred. Both cases have also been considered by the authors of the Swedish outbreak who found similar clonal heterogeneity within single cows as well as Single Nucleotide Variants (SNV: SNP + single nucleotide indels) that exhibited population penetrance below the detection level [50]. In the study at hand, SNPs 1–5 were observed to be isolate specific within the investigated population of 114 tested isolates (34 for site A and 80 for site C). Regarding the whole population in the respective burial site, the odds for a unique SNP to occur in any specific isolate are very low because this variation must have originated from an error during the last round(s) of DNA-replication associated with cell division before sporulation, i.e., present only in one or only a few bacteria of the entire population within the dying animal.

It is noteworthy, that the authors of the Swedish outbreak-investigation [50] found specific SNVs for every isolate, even between those from a single host animal or soil samples taken from near-by. This phenomenon of a higher mutation rate compared to the Pollino sites can most easily be explained by the selective pressure applied during antibiotic treatment of the infected Swedish bovines. No similar selective pressure was present at the burial-sites in Pollino.

Regarding the isolate specific SNPs 1–5, it is striking that the ratio of SNPs found near-carcass to near-surface is 1 to 4. This higher number of isolate specific SNPs in near-surface soil may be the result of growth and replication of *B*. *anthracis*. This notion is strongly supported by the finding that the pXO1-negative isolate C3/5 cm– 1 harbors one of these SNPs (SNP 4). However, as aforementioned, it is very likely that the SNPs, which were found to be isolate-specific within this study, are present in other not-yet isolated endospores in soil of the respective burial sites. Under the assumption of ongoing soil proliferation, it has to be expected that these clonal strains can be found in the immediate vicinity, *e*.*g*. in the same soil sample, as the isolates harboring the SNPs. Because of the enormous number of endospores present in these soils, not every possible isolate had been cultured. Therefore, SNPs 1 and 5, which were both present in C1/5 cm– 2, were not detected in one of the other 16 isolates of soil sample C1/5 cm but still might be more abundant at this location. In the light of this aspect, emergence of these mutations during late phases of infection with a subsequent dispersal in soil seems also plausible. However, ongoing multiplication in soil would probably lead to a population of vegetative cells potentially containing the observed SNPs as well. These vegetative cells, if existing, would not be captured with the GABRI (or any other current *B*. *anthracis*-isolation) method because thermal inactivation of vegetative growth is part of any extant isolation procedure. Thus, to give a clear-cut answer on the question whether these SNPs are the result of near-surface soil replication or simply accrue in the course of infection, a broader spectrum of strains would have to be analyzed for SNP distribution. As applied previously [50] investigating penetrance of SNPs in the whole population would be even better, but yields of *B*. *anthracis* DNA from endospores in soil samples were very poor (data not shown). A possible solution for this challenge would be to introduce an enrichment step in liquid medium selective for *B*. *anthracis* directly from soils prior to inactivation and DNA extraction. This mixed-population DNA might then be further analyzed for the presence of SNP-markers by quantitative methods such as digital PCR which would numerically tell the percentage of ancestral or derived allele, respectively [51].

## Conclusion

We hypothesized that a soil-borne life cycle of *B*. *anthracis* exists at anthrax burial sites leading to microevolution increasing the genetic diversity from the depth of the carcass to the near-surface. In this study, there was no clear-cut higher overall genetic diversity of *B*. *anthracis* in near-surface than in near-carcass isolates. Variations revealed by MLVA and SNR analysis are most likely due to mutation events during host infection. Conversely, the distribution of isolate-specific SNPs, obtained by the technique yielding the highest resolution power, i.e., whole genome sequencing, together with the recognition of loss of pXO1 in a near-surface isolate, clearly suggest a soil-borne life cycle of *B*. *anthracis*.

**Fig 4:**
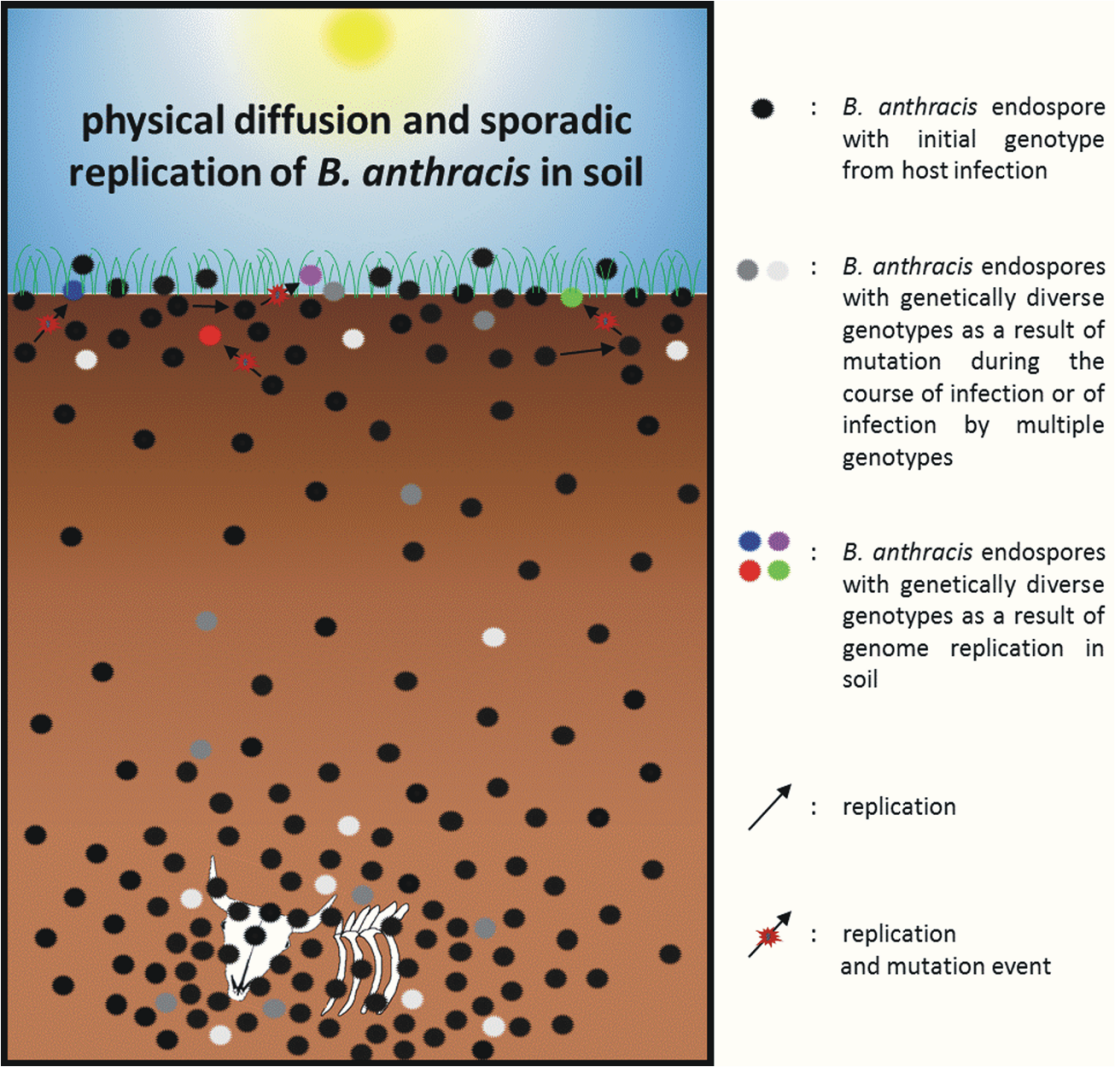
Modified (synthesis) model for *B*. *anthracis* genotype dynamics in soil of Pollino burial sites. Soil surrounding the carcass at the lower part of the figure harbors a very high endospore burden. Genetically diverse genotypes, which can be found near-carcass and near-surface, are the result of either multiple genotype infection or mutations during the course of host infection. Endospores reach the surface and accumulate via physical diffusion. Sporadic germination in soil or possibly in transient vectors, replication and sporulation under favorable conditions can lead to genetically diverse genotypes that can be found in near-surface soil.

As a consequence of these results, massive proliferation, as depicted in Fig 1, is probably not supported in soils of Pollino burial sites. Therefore, a proposed significant multiplication posing a risk for infection can be all but eliminated as the reason for spore accumulations at the surface. However, finding a SNP containing pXO1-negative strain and a strain harboring two SNPs in near-surface soil of burial site C indicates that at least some or limited soil replication takes place in at least one of the two burial sites. Hence, we propose a modified model for *B*. *anthracis* soil population dynamics combining both hypothesis and antithesis (Fig 1) to a final synthesis (Fig 4), which states physical diffusion to be the cause of local accumulations of endospores at or near the surface where only sporadic soil replication takes place under favorable conditions, possibly within the rhizosphere or in soil-dwelling protists as proposed earlier [27,28]. In contrast, it is hitherto unknown how much proliferation of *B*. *anthracis* occurs at deeper soil layers beneath carcass sites. Until this question is experimentally addressed we have to assume that most of the activity takes place within the upper 25 cm of soil horizons where most active plant-, algal-, protist- and small animal-life is found [35]. At contaminated sites, limited proliferation of *B*. *anthracis* might even result in interaction with grasses such as *Enneapogon desvauxii*, promoting the pathogens temporal establishment, offering a potential disease transmission route to large grazing animal hosts [62].

Although an unambiguous explanation cannot be provided for the obscure processes of *B*. *anthracis* in soil environments, this study was the first to address this issue *in situ*. For further genomic investigations on the fate of *B*. *anthracis* in soil, it would be most favorable to study the developmental progression of anthrax burial sites over many years beginning with the thorough analysis of strains isolated directly from the diseased animal in order to compare the genotypes of these organisms with those of isolates collected after fixed-time intervals from contaminated soil. To obtain reliable information on the genetic diversity at such sites and thus, detect potential changes, it would be crucial to test a sufficiently large subpopulation of the monomorphic pathogen by massive whole genome sequencing which might be an easy feat in the near future. Apart from a genomic approach, the unprecedented detection of the elusive vegetative forms of *B*. *anthracis* in soil environments would clearly bring the existence of soil-borne life cycle in *B*. *anthracis* to the light of the day.

## Supporting Information

S1 Fig On-site burial at high-altitude pasture of a diseased cow from the 2004 anthrax outbreak at Pollino National Park. (TIF)

## References

[1] Turnbull PC (2008) World Health Organization. Anthrax in humans and animals In: (Editor) PT, editor. Geneva (CH): WHO Press.

[2] Ardi P, Emanuele C, Luigina S, Leonardo M, Jonida B, Bizena B, et al (2015) Genotyping of *Bacillus anthracis* strains circulating in Albania. Journal of Bioterrorism & Biodefense 7: 1–6.

[3] Okello A, Welburn S, Smith J (2014) Crossing institutional boundaries: mapping the policy process for improved control of endemic and neglected zoonoses in sub-Saharan Africa. Health Policy Plan. 10.1093/heapol/czu059 25000963

[4] Chakraborty A, Khan SU, Hasnat MA, Parveen S, Islam MS, Mikolon A, et al (2012) Anthrax outbreaks in Bangladesh, 2009–2010. Am J Trop Med Hyg 86: 703–710. 10.4269/ajtmh.2012.11-0234 22492157 PMC3403762

[5] Hugh-Jones M (1999) 1996–97 global anthrax report. J Appl Microbiol 87: 189–191. 10475945 10.1046/j.1365-2672.1999.00867.x

[6] Léonard C, Chen Y, Mahillon J (1997) Diversity and differential distribution of IS231, IS232 and IS240 among *Bacillus cereus*, *Bacillus thuringiensis* and *Bacillus mycoides* . Microbiology 143 (Pt 8): 2537–2547. 9274007 10.1099/00221287-143-8-2537

[7] Inglesby TV, O'Toole T, Henderson DA, Bartlett JG, Ascher MS, Eitzen E, et al (2002) Anthrax as a biological weapon, 2002: updated recommendations for management. JAMA 287: 2236–2252. 11980524 10.1001/jama.287.17.2236

[8] Fowler RA, Shafazand S (2011) Anthrax bioterrorism: prevention, diagnosis and management strategies. J Bioterr Biodef 2: 10.4172/2157-2526.1000107

[9] Keim P, Van Ert MN, Pearson T, Vogler AJ, Huynh LY, Wagner DM (2004) Anthrax molecular epidemiology and forensics: using the appropriate marker for different evolutionary scales. Infect Genet Evol 4: 205–213. 15450200 10.1016/j.meegid.2004.02.005

[10] Van Ert MN, Easterday WR, Huynh LY, Okinaka RT, Hugh-Jones ME, Ravel J, et al (2007) Global genetic population structure of *Bacillus anthracis* . PLoS One 2: e461 17520020 10.1371/journal.pone.0000461 PMC1866244

[11] Marston CK, Allen CA, Beaudry J, Price EP, Wolken SR, Pearson T, et al (2011) Molecular epidemiology of anthrax cases associated with recreational use of animal hides and yarn in the United States. PLoS One 6: e28274 10.1371/journal.pone.0028274 22174783 PMC3235112

[12] Pearson T (2004) Phylogenetic discovery bias in *Bacillus anthracis* using single-nucleotide polymorphisms from whole-genome sequencing. Proceedings of the National Academy of Sciences 101: 13536–13541. 10.1073/pnas.0403844101 PMC518758 15347815

[13] Kenefic LJ, Beaudry J, Trim C, Huynh L, Zanecki S, Matthews M, et al (2008) A high resolution four-locus multiplex single nucleotide repeat (SNR) genotyping system in *Bacillus anthracis* . Journal of Microbiological Methods 73: 269–272. 10.1016/j.mimet.2007.11.014 18237793

[14] Keim P, Gruendike JM, Klevytska AM, Schupp JM, Challacombe J, Okinaka R (2009) The genome and variation of *Bacillus anthracis* . Molecular Aspects of Medicine 30: 397–405. 10.1016/j.mam.2009.08.005 19729033 PMC3034159

[15] Hoffmaster AR, Fitzgerald CC, Ribot E, Mayer LW, Popovic T (2002) Molecular subtyping of *Bacillus anthracis* and the 2001 bioterrorism-associated anthrax outbreak, United States. Emerg Infect Dis 8: 1111–1116. 12396925 10.3201/eid0810.020394 PMC2730295

[16] Hugh-Jones M, Blackburn J (2009) The ecology of *Bacillus anthracis* . Molecular Aspects of Medicine 30: 356–367. 10.1016/j.mam.2009.08.003 19720074

[17] Bellan SE, Turnbull PC, Beyer W, Getz WM (2013) Effects of experimental exclusion of scavengers from carcasses of anthrax-infected herbivores on *Bacillus anthracis* sporulation, survival, and distribution. Appl Environ Microbiol 79: 3756–3761. 10.1128/AEM.00181-13 23584788 PMC3675950

[18] Fasanella A, Van Ert M, Altamura SA, Garofolo G, Buonavoglia C, Leori G, et al (2005) Molecular Diversity of *Bacillus anthracis* in Italy. Journal of Clinical Microbiology 43: 3398–3401. 16000465 10.1128/JCM.43.7.3398-3401.2005 PMC1169099

[19] Garofolo G, Ciammaruconi A, Fasanella A, Scasciamacchia S, Adone R, Pittiglio V, et al (2010) SNR analysis: molecular investigation of an anthrax epidemic. BMC Veterinary Research 6: 11 10.1186/1746-6148-6-11 20187980 PMC2837646

[20] Van Ness GB (1971) Ecology of anthrax. Science 172: 1303–1307. 4996306 10.1126/science.172.3990.1303

[21] Girault G, Parisot N, Peyretaillade E, Peyret P, Derzelle S (2014) Draft genomes of three strains representative of the *Bacillus anthracis* diversity found in France. Genome Announc 2. 10.1128/genomeA.00736-14 PMC4118061 25081258

[22] Brahmbhatt TN, Janes BK, Stibitz ES, Darnell SC, Sanz P, Rasmussen SB, et al (2007) *Bacillus anthracis* exosporium protein BclA affects spore germination, interaction with extracellular matrix proteins, and hydrophobicity. Infect Immun 75: 5233–5239. 17709408 10.1128/IAI.00660-07 PMC2168272

[23] Sterne M (1959) Anthrax In: Stableforth AW, Galloway IA, editors. Infectious Diseases of Animals, Disease due to Bacteria. London: Butterworth pp. 16–52.

[24] Jensen GB, Hansen BM, Eilenberg J, Mahillon J (2003) The hidden lifestyles of *Bacillus cereus* and relatives. Environ Microbiol 5: 631–640. 12871230 10.1046/j.1462-2920.2003.00461.x

[25] Halverson L, Clayton M, Handelsman J (1993) Population biology of *Bacillus cereus* UW85 in the rhizosphere of field-grown soybeans. Soil Biol Biochem 25: 485–494.

[26] Vilain S, Luo Y, Hildreth MB, Brozel VS (2006) Analysis of the life cycle of the soil saprophyte *Bacillus cereus* in liquid soil extract and in soil. Appl Environ Microbiol 72: 4970–4977. 16820495 10.1128/AEM.03076-05 PMC1489341

[27] Saile E, Koehler TM (2006) *Bacillus anthracis* multiplication, persistence, and genetic exchange in the rhizosphere of grass plants. Appl Environ Microbiol 72: 3168–3174. 16672454 10.1128/AEM.72.5.3168-3174.2006 PMC1472387

[28] Dey R, Hoffman PS, Glomski IJ (2012) Germination and amplification of anthrax spores by soil-dwelling amoebas. Appl Environ Microbiol 78: 8075–8081. 10.1128/AEM.02034-12 22983962 PMC3485947

[29] Minett F.C., Dhanda M.R. (1941) Multiplication of *B*. *anthracis* and *Cl*. *chauvoei* in soil and water. Indian Journal of Veterinary Science and Animal Husbandry 11: 308–321.

[30] Schuch R, Fischetti VA (2009) The secret life of the anthrax agent *Bacillus anthracis*: bacteriophage-mediated ecological adaptations. PLoS One 4: e6532 10.1371/journal.pone.0006532 19672290 PMC2716549

[31] Smart KF, Aggio RB, Van Houtte JR, Villas-Boas SG (2010) Analytical platform for metabolome analysis of microbial cells using methyl chloroformate derivatization followed by gas chromatography-mass spectrometry. Nat Protoc 5: 1709–1729. 10.1038/nprot.2010.108 20885382

[32] Antwerpen M, Ilin D, Georgieva E, Meyer H, Savov E, Frangoulidis D (2011) MLVA and SNP analysis identified a unique genetic cluster in Bulgarian *Bacillus anthracis* strains. European Journal of Clinical Microbiology & Infectious Diseases 30: 923–930. 10.1007/s10096-011-1177-2 21279731

[33] Aikembayev AM (2010) Historical distribution and molecular diversity of *Bacillus anthracis*, Kazakhstan. Emerging Infectious Diseases. 10.3201/eid1605.091427 PMC2953997 20409368

[34] Turnbull PCB, Hutson RA, Ward MJ, Jones MN, Quinn CP, Finnie NJ, et al (1992) *Bacillus anthracis* but not always anthrax. Journal of Applied Bacteriology 72: 21–28. 1541596 10.1111/j.1365-2672.1992.tb04876.x

[35] Fierer N, Schimel JP, Holden PA (2003) Variations in microbial community composition through two soil depth profiles. Soil Biology and Biochemistry 35: 167–176.

[36] Fasanella A, Di Taranto P, Garofolo G, Colao V, Marino L, Buonavoglia D, et al (2013) Ground Anthrax *Bacillus* Refined Isolation (GABRI) method for analyzing environmental samples with low levels of *Bacillus anthracis* contamination. BMC Microbiol 13: 167 10.1186/1471-2180-13-167 23865983 PMC3728113

[37] Jacob D, Sauer U, Housley R, Washington C, Sannes-Lowery K, Ecker DJ, et al (2012) Rapid and high-throughput detection of highly pathogenic bacteria by Ibis PLEX-ID technology. PLoS ONE 7: e39928 10.1371/journal.pone.0039928 22768173 PMC3386907

[38] Antwerpen MH, Zimmermann P, Bewley K, Frangoulidis D, Meyer H (2008) Real-time PCR system targeting a chromosomal marker specific for *Bacillus anthracis* . Mol Cell Probes 22: 313–315. 10.1016/j.mcp.2008.06.001 18602986

[39] Birdsell DN, Pearson T, Price EP, Hornstra HM, Nera RD, Stone N, et al (2012) Melt analysis of mismatch amplification mutation assays (Melt-MAMA): a functional study of a cost-effective SNP genotyping assay in bacterial models. PLoS ONE 7: e32866 10.1371/journal.pone.0032866 22438886 PMC3306377

[40] Beyer W, Bellan S, Eberle G, Ganz HH, Getz WM, Haumacher R, et al (2012) Distribution and molecular evolution of *Bacillus anthracis* genotypes in Namibia. PLoS Negl Trop Dis 6: e1534 10.1371/journal.pntd.0001534 22413024 PMC3295808

[41] Keim P, Price LB, Klevytska AM, Smith KL, Schupp JM, Okinaka R, et al (2000) Multiple-locus variable-number tandem repeat analysis reveals genetic relationships within *Bacillus anthracis* . J Bacteriol 182: 2928–2936. 10781564 10.1128/jb.182.10.2928-2936.2000 PMC102004

[42] Lista F, Faggioni G, Valjevac S, Ciammaruconi A, Vaissaire J, le Doujet C, et al (2006) Genotyping of *Bacillus anthracis* strains based on automated capillary 25-loci multiple locus variable-number tandem repeats analysis. BMC Microbiol 6: 33 16600037 10.1186/1471-2180-6-33 PMC1479350

[43] Kenefic LJ, Beaudry J, Trim C, Daly R, Parmar R, Zanecki S, et al (2008) High resolution genotyping of *Bacillus anthracis* outbreak strains using four highly mutable single nucleotide repeat markers. Letters in Applied Microbiology 46: 600–603. 10.1111/j.1472-765X.2008.02353.x 18363651

[44] Stratilo CW, Bader DE (2012) Genetic diversity among *Bacillus anthracis* soil isolates at fine geographic scales. Applied and Environmental Microbiology 78: 6433–6437. 10.1128/AEM.01036-12 22773624 PMC3426704

[45] Langmead B, Salzberg SL (2012) Fast gapped-read alignment with Bowtie 2. Nat Methods 9: 357–359. 10.1038/nmeth.1923 22388286 PMC3322381

[46] Li H, Handsaker B, Wysoker A, Fennell T, Ruan J, Homer N, et al (2009) The sequence alignment/map format and SAMtools. Bioinformatics 25: 2078–2079. 10.1093/bioinformatics/btp352 19505943 PMC2723002

[47] Milne I, Stephen G, Bayer M, Cock PJ, Pritchard L, Cardle L, et al (2013) Using Tablet for visual exploration of second-generation sequencing data. Brief Bioinform 14: 193–202. 10.1093/bib/bbs012 22445902

[48] Fasanella A, Braun P, Grass G, Hanczaruk M, Aceti A, Serrecchia L, et al (2015) Genome sequence of *Bacillus anthracis* isolated from an anthrax burial site in Pollino National Park, Basilicata region (southern Italy). Genome Announc 3. 10.1128/genomeA.00141-15 PMC4395053 25792059

[49] Ye J, Coulouris G, Zaretskaya I, Cutcutache I, Rozen S, Madden TL (2012) Primer-BLAST: a tool to design target-specific primers for polymerase chain reaction. BMC Bioinformatics 13: 134 10.1186/1471-2105-13-134 22708584 PMC3412702

[50] Ågren J, Finn M, Bengtsson B, Segerman B (2014) Microevolution during an anthrax outbreak leading to clonal heterogeneity and penicillin resistance. PLoS One 9: e89112 10.1371/journal.pone.0089112 24551231 PMC3923885

[51] Straub T, Baird C, Bartholomew RA, Colburn H, Seiner D, Victry K, et al (2013) Estimated copy number of *Bacillus anthracis* plasmids pXO1 and pXO2 using digital PCR. J Microbiol Methods 92: 9–10. 10.1016/j.mimet.2012.10.013 23142659

[52] Okinaka RT, Cloud K, Hampton O, Hoffmaster AR, Hill KK, Keim P, et al (1999) Sequence and organization of pXO1, the large *Bacillus anthracis* plasmid harboring the anthrax toxin genes. J Bacteriol 181: 6509–6515. 10515943 10.1128/jb.181.20.6509-6515.1999 PMC103788

[53] Vogler AJ, Keys C, Nemoto Y, Colman RE, Jay Z, Keim P (2006) Effect of repeat copy number on variable-number tandem repeat mutations in *Escherichia coli* O157:H7. J Bacteriol 188: 4253–4263. 16740932 10.1128/JB.00001-06 PMC1482962

[54] Beyer W, Turnbull PC (2013) Co-infection of an animal with more than one genotype can occur in anthrax. Lett Appl Microbiol. 10.1111/lam.12140 23937393

[55] Johansen TB, Agdestein A, Lium B, Jorgensen A, Djonne B (2014) *Mycobacterium avium* subsp. hominissuis infection in swine associated with peat used for bedding. Biomed Res Int 2014: 189649 10.1155/2014/189649 25431762 PMC4241287

[56] Stratilo CW, Lewis CT, Bryden L, Mulvey MR, Bader D (2006) Single-nucleotide repeat analysis for subtyping *Bacillus anthracis* isolates. J Clin Microbiol 44: 777–782. 16517854 10.1128/JCM.44.3.777-782.2006 PMC1393151

[57] Hahn KR, Janzen TW, Thomas MC, Shields MJ, Goji N, Valle E, et al (2014) Single nucleotide repeat analysis of *B*. *anthracis* isolates in Canada through comparison of pyrosequencing and Sanger sequencing. Vet Microbiol 169: 228–232. 10.1016/j.vetmic.2013.12.020 24485934

[58] Quail MA, Smith M, Coupland P, Otto TD, Harris SR, Connor TR, et al (2012) A tale of three next generation sequencing platforms: comparison of Ion Torrent, Pacific Biosciences and Illumina MiSeq sequencers. BMC Genomics 13: 341 10.1186/1471-2164-13-341 22827831 PMC3431227

[59] Price EP, Seymour ML, Sarovich DS, Latham J, Wolken SR, Mason J, et al (2012) Molecular epidemiologic investigation of an anthrax outbreak among heroin users, Europe. Emerging Infectious Diseases 18: 1307–1313. 10.3201/eid1808.111343 22840345 PMC3414016

[60] Kupferschmidt K (2011) Epidemiology. Outbreak detectives embrace the genome era. Science 333: 1818–1819. 10.1126/science.333.6051.1818 21960605

[61] Liu L, Li Y, Li S, Hu N, He Y, Pong R, et al (2012) Comparison of next-generation sequencing systems. Journal of Biomedicine and Biotechnology 2012: 1–11. 22829749 10.1155/2012/251364 PMC3398667

[62] Ganz HH, Turner WC, Brodie EL, Kusters M, Shi Y, Sibanda H, et al (2014) Interactions between *Bacillus anthracis* and plants may promote anthrax transmission. PLoS Negl Trop Dis 8: e2903 10.1371/journal.pntd.0002903 24901846 PMC4046938

