# Supplementary figures and images for: Microevolution of Anthrax from a Young Ancestor (M.A.Y.A.) Suggests a Soil-Borne Life Cycle of Bacillus anthracis
Source: PLoS One. 2015 Aug 12;10(8):e0135346. doi: 10.1371/journal.pone.0135346 (PMC4534099; doi:10.1371/journal.pone.0135346)

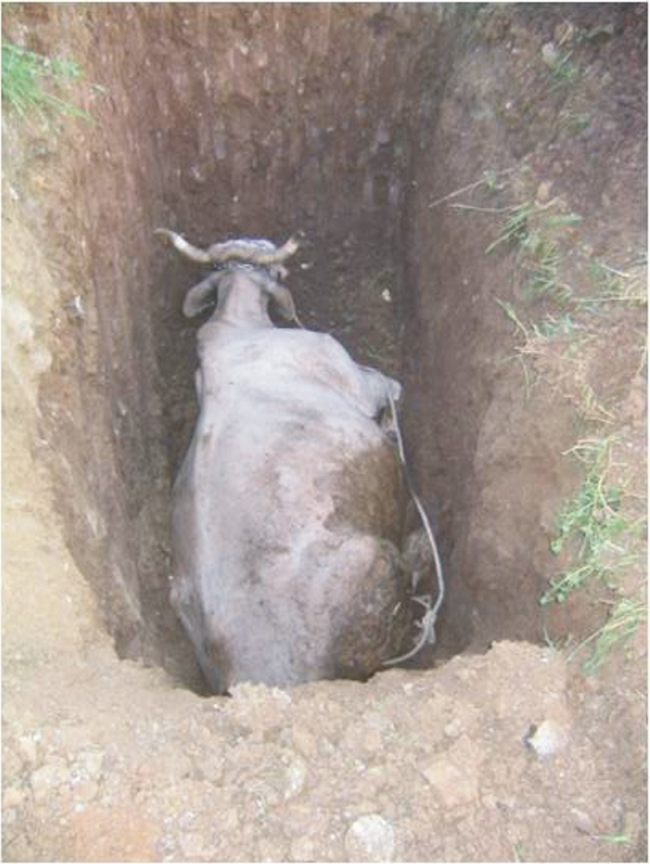

Supplement: S1 Fig — (TIF) [file pone.0135346.s001.tif]
